# Aggregate Shocks and Enrollment Rates over a Decade in Malawi

**DOI:** 10.21203/rs.3.rs-3927724/v1

**Published:** 2024-02-08

**Authors:** Asma Hyder

**Affiliations:** Institute of Business Administration Karachi

## Abstract

In this paper we report the impact of shocks of different nature and their impact on child schooling. The study is based on *Malawi Longitudinal Study of Families and Health (MLSFH)* project, which provide information on seven different types of shocks and along with information on schooling of boys and girls from 2008 to 2019. We found that all shocks climatic nature or otherwise have negative effect on child enrollment except in case of damage to the house substitution effect dominate and we found evidence that girls of 7–12 years of age and boys in 13–17 years of age cohort have positive correlation with this specific shock. We also found that frequency of two shocks i.e., poor crop yield and price shock have significantly deceased as compared to other types of shocks over last 10 years. However, unemployment, business failure, divorces and damages to houses remain quite persistent over last decade. Study support suggest the social protection programs for children to address the vulnerabilities faced by households of rural Malawi.

## Introduction

Extreme weather risk is very high in Sub-Saharan Africa and droughts are one the most frequent shock among others (World Bank 2014). Due to high frequency of extreme climatic events the food prices and input prices are very unstable; thus, households have to face direct and indirect impact of such events. Beside these weather shocks the HIV/AIDS and low life expectancy is another shock which have negative impact on households’ wellbeing. The coping mechanism during and after crisis is very complex and number of studies address to understand this mechanism. Amongst those different coping mechanisms one popular mechanism to increase the labor capacity and to reduce the opportunity cost is drop out of children from schools. The understanding of such behavior need a study spanning over a longer period of time, thus this paper attempt to study this behavior over a decade in rural Malawi.

The exposure to adverse events in underdeveloped countries disrupt the family stability in the form of continuity and predictability in the life of children (Ackerman, Kogos, Youngstrom, Schoff, & Izard, 1999) especially in schooling. In many poor societies the households depend on agriculture to support their families, mostly this dependance on agriculture is in the form of subsistence farming. The agriculture yield and demand for agricultural products is very volatile and social safety nets are very weak in developing countries (Jensen 2000) thus may cause negative effect on households’ welfare. The agricultural productive in context of Africa, slightly increased in recent years but still it remains very low as compared to work developing or even underdeveloped countries of the world (Collier and Dercon 2014). The agriculture productivity in this region heavily depends on rainfall (Hansen et al. (2011)) and also adversely affected by extreme weather events (Dercon & Christiaensen 2011).

Investment in children is a critical and important decision for the households and highly correlated with their income flows. The negative shocks may affect adversely the investment in education of their children (See for instance, Skoufias (1997), Foster (1995), and Duryea)). In response of coping any negative economic shock, especially in developing countries the most popular option is taking children out of school (Beegle, Dehejia, and Gatti, 2005; Sawada, 2003). Nevertheless, few researchers presented evidence on substitution and income effect, which is helpful in designing intervention during or after shock period (Hyder (2015) and Ferreira and Schady (2009)). The income and substitution effects may dominate over each other depending on type of shock, age, and gender of the child. Depending on dynamics of shocks the outcomes are different for boys and girls and for each age group. Shocks, which reduces the labor market opportunities may not affect the enrollment of older cohort of boys. However, the price shock may affect boys’ enrollment adversely. Similarly, the death of caregiver may affect the younger cohort with ambiguous gender-wise effects depending upon the social safety nets and vice versa.

Malawi is a land locked country in southeastern part of Africa with the population of approximately 18.6 million and gender ratio (men per hundred women) is 98[1]. The approximate area of the country is 118000 KM^2^. The proportion of the population under the age of 15 is 47% and above 60 years is 5%, which shows the low life expectancy and larger younger population. The literacy rate is 75.5% for men and 48.7% for women (WHO 2005). The country is largely dependent on agriculture. The frequency of climatic extreme events often reduces the welfare of households due to negative impact on farms and industries. Lake Malawi is significant as it is a source of livelihood for many households beside it also take one fifth of the country’s area (Baquie and Habtamu (2020)). The country is divided into three regions, i.e., Mchinji (in the Central), Balaka (in the Southern) and Rumphi (in the Northern). Malawi is considered as one of the world’s poorest countries. In terms of HDI, Malawi is ranked at 174^th^ position in 2019 (HDR 2020) and its GDP is 25.037 million dollars if measured in PPP (IMF 2021). According to World Bank (2016) 95% Malawian poor population live in rural areas and over all the southern region has the highest proportion of ultra-poor. World bank’s Malawi Economic Monitor (MEM 2022) claims that a series of external and domestic shocks are putting pressure on Malawi’s macro-economy, increasing the urgency to protect essential services for the vulnerable population of the country.

Novelty of this study lies in the unique nature of measurement of shocks, i.e., self-reported shocks. We use six different types of self-reported shocks to examine their impact on schooling of boys and girls, thus provide evidence of the differences in impact of different shock on each gender. Another, distinguishing feature is continuous nature of the shock variables. The all six shocks appear as proportion of households those who have reported them as a shock in a certain village. The higher the proportion- the more that village is at risk on respective shock and thus the vulnerability is high in that village.

## Data

This paper exploits data from *Malawi Longitudinal Study of Families and Health (MLSFH)* project. The MLSFH is one of a very few long-standing longitudinal cohort studies in a poor sub-Saharan African (SSA) context. It provides a rare record of more than a decade of demographic, socioeconomic and health conditions in one of the poorest socies of the world.[2] The sample characteristics of this survey are very close to those Demographic Health Survey (DHS) for rural Malawi (Anglewicz et al. 2009). The MLSFH represent rural Malawi and data is collected from Malawian households representing rural areas which are similar to the rural areas of the similar countries.

The panel data is at village level, which include 124 villages across three regions of rural Malawi. There are 65 villages in Mchinji (central), 17 villages in Balaka (southern) and 42 villages in Rumphi (northern). We utilize four waves of data from 2008, 2010, 2017 and 2019. The shock variables used in table 3–6 are village level proportion of households experienced shocks in each year in their respective villages. Similarly, the four dependent variables are the proportion of boys and girls enrolled in school in their specific age cohort.

The summary statistics of proportion of households experienced these shocks within their respective village show that shock of prices of agriculture input and output significantly controlled over one decade. The last column, which shows the difference between 2008 and 2019, present the difference of proportion of effected households from a particular shock. The significant reduction in self-reported agriculture price shock reflects two possibilities. First, that government controlled the prices of agricultural input or output product from being volatile and thus protected the rural households to some extent; second, the households in rural Malawi develop mechanism to buffer price shock related to agricultural prices. The later possibility is more plausible as households are now better prepared for consumption smoothing. Household shocks like damage to the house or breakup in family remain unchanged over the period under consideration.

The improvement in enrollment rate of four age cohorts almost remain the same with southern region on lead. The stagnant rather decreasing trend is girls in older cohort, i.e., 13–17 years the enrollment rates slightly reduced are thus worrying.

## Analysis and Discussion

The decision-making regarding investment in children during the time of crisis is considerably different from normal time. The type of shock and gender of the child are main factors which parents consider making such decisions. Especially when social net or mechanism to buffer stocks are weak in society.[3] The literature suggests broadly three channels affecting child wellbeing in case of hitting an adverse shock to the household (Case et al, (2004) and Gartler et al, (2004), and Bell et al, (2006)). First. The direct effect due to loss in income especially in absence of credit and insurance market failure- a very common phenomena in developing countries. Secondly, the preferences regarding the investment in children may change due to new power structure or alternate caregiver options. Finally, the child wellbeing is adversely affected due to psychological factors after facing the adverse event/ shock. We analyze the impact of shock for both genders separately, given that gender inequality plays a major role in causing and perpetuating poverty. The gender biases are not limited to adults, rather those biases are visible during initial years of life (Hillman and Jenkner 2004).

For this analysis we used fixed effect model on village panel. The results are presented in table 3–6 for four demographic groups. The death of the bread earner (shock 1) is the most devastating shock in case on both cohorts of boys schooling. The life expectancy is 40 years in Malawi both for men and women as reported in Kauye and Mafuta (2007). There is ample empirical evidence that death of any one or both parents have a significant impact of child wellbeing and especially in context of Africa (Case 2004), these phenomena become even more important because of high number of orphans and with absence of any social safety nets. Although shock 1 in this study don’t mention clearly that this is death of parent but usually father is the main bread earner in Malawi like many other patriarchal or underdeveloped societies. This result is also supported by evolutionary biology and hypothesis by Hamilton (1964a, 1964b) which state that degree of relatedness decreases/increases with distance of genetic relationship. The effect of caregiver’s death has ambiguous effects on child schooling (Paul 2004). However, we find that in case of Malawi, the death of father or caregiver, who is the main source of income, have significant negative effect on boys. Thus, the direct effect of loss of income results in drop out of boys from school in rural Malawi. The absence of insurance and security nets from Government is also responsible for this loss in boys’ education during the family crisis. In such cases the additional benefits may reduce the household reliance on child labor and would be helpful for continuation of children continuation in schooling (Ravallion and wodon (1999); Grootaert and Kanbur (1995)). We couldn’t establish any relationship between girls’ enrollment and death of bread earner in household. The girls’ participation in labor market is low in most of the underdeveloped countries and their participation in household chores is considered more beneficial as compared to income generation activities. There are some papers claim that channel of this particular outcome (for instance, Thomas (1990), Haddad, Hoddinott, and Alderman (1997)), i.e., drop out of boys’ and girls’ enrollment remain intact is due to change in power structure. In case of death of father (care giver), if mother become responsible, she more likely decides to send boys to market and for girls the routine remains the same. However, we don’t explore the gender of care giver and change of dynamics of the power structure of the death of care giver.

The frequency of shocks related to agricultural products like poor crop yield, loss of crops due to diseases or pests or the loss of livestock although reduced and there is no significant effect of this shock on schooling of all cohorts of boys and girls. As discussed in few other studies that response of shocks in terms of enrollments is hard to define due to nature of the shock. Also, due to crop failure, the larger possibility is that demand for work in fields will not increase particularly in rural areas. Secondly, the power structure of the household remains same thus the preferences regarding schooling remain the same before and after crop failure shock. Considering that basic schooling is provided by school thus this shock don’t pushes students of either gender out of schools.

Loss of source of income—such as loss of employment, business failure, someone who had been assisting the household stopped their support remain almost same throughout the ten years under consideration (See table 1). We found that only girls 7–12 years of age are negatively and significantly affected by this shock. The consistency in frequency shows that households probably consider this a routine matter and prepared to continue their routine life. Only younger cohort of girls are dropped out probable channel as copying strategy or due to marginal investment had been made on younger girls. However, few studies found different results against the rainfall shocks but for older cohort of girls in Uganda (Martina 2013)

One of the noteworthy results is negative impact of household break-up like divorce of parents on younger cohort of both boys and girls. Children living in broken/ distracted families, or children living with stepparents or without parents, get less inspiration from their parents, less attention in terms of emphasize on studies, both psychological and economic pressures, and due to many other factors, they have significant negative impact on schooling. For instance, many studies in literature, including, Amato (1987); Furstenberg and Nord (1985); Furstenberg, Nord, Peterson, and Zill (1983) and Nock (1988); Steinberg (1987) are helpful to understand different channels to examine this impact. The data shows that incidence of family break up reduced but in an insignificant proportion and specially the event of family break up seems almost unchanged as compared to other shocks, which are reduced comparatively in better proportion. Over time, changing nuptiality due to HIV/AIDS, climatic and economic vulnerabilities in Sub-Saharan Africa is a concern for demographers (Dintwa, 2010; Mokomane, (2013), Wusu & Isiugo-Abanihe, 2006).

## Conclusion

This study concludes the impact of shocks of different nature and their impact on child schooling. With the help of *Malawi Longitudinal Study of Families and Health (MLSFH)* project, which provide information on seven different types of shocks and along with information on schooling of boys and girls from 2008 to 2019. We found that all shocks climatic nature or otherwise have negative effect on child enrollment except in case of damage to the house substitution effect dominate and we found evidence that girls of 7–12 years of age and boys in 13–17 years of age cohort have positive correlation with this specific shock. The study supports the argument of Carmona (2009) that parents experience of poverty in the form of education, food insecurity or psychological are transmitted to next generation. We also found that frequency of two shocks i.e., poor crop yield and price shock have significantly deceased as compared to other types of shocks over last 10 years. However, unemployment, business failure, divorces and damages to houses remain quite persistent over last decade. Thus, school enrollment is very sensitive in response of shocks due to few governance problems. At many places parents are expected to contribute labor and material to school construction and to buy school supplies and clothes. Thus, parents when calculate the cost of schooling along with foregone child labor, the cost-benefit analysis is not in favor of schooling (Hillman and Jenkner 2004). This practice increases the vulnerability for attending school.

The article suggests for the development of social protection mechanism for all cohorts of children in rural areas of Malawi to help mitigating the impact of adverse events, whether climatic, economic or within the household like destruction within households, especially from the perspective of girls. The study also suggests and identifies the need for public policy to provide a buffer mechanism against adverse events and specially to reduce the intergenerational transmission of negative shocks. Child support grants and social security programs, both for girls and boys of different cohorts must be provided to avoid intergenerational mobility of poverty and to mitigate the household vulnerabilities.

There are few other mechanisms to response the adverse shocks including changing school type like prom private to public or any other type of school, which might an interesting future agenda. Also, in few developing countries private schools provide different options to parents which help them smoothing the household consumption without dropping out of their children from schools (Kingdon, 2007, 2017; Muralidharan and Kremer, 2009). Such type of incentives are required in countries like Malawi and an interesting future research agenda for future research.

## Figures and Tables

**Table 1:** Summary Statistics of Shocks over Time and Region

	Year	2008		2010		2017		2019		Diff in mean
Shock	Region	Mean	S.D	Mean	S.D	Mean	S.D	Mean	S.D	2008–2019
**Shock 1**	**Central**	0.52	0.5	0.4	0.49	0.48	0.5	0.25	0.43	−0.27
	**Southern**	0.42	0.49	0.37	0.48	0.4	0.49	0.17	0.38	−0.25
	**Northern**	0.36	0.48	0.33	0.47	0.41	0.49	0.24	0.43	−0.12
**Shock 2**	**Central**	0.74	0.44	0.72	0.45	0.65	0.48	0.51	0.5	−0.23
	**Southern**	0.86	0.35	0.72	0.45	0.88	0.32	0.55	0.5	−0.31
	**Northern**	0.6	0.49	0.5	0.5	0.78	0.41	0.48	0.5	−0.12
**Shock 3**	**Central**	0.4	0.49	0.46	0.5	0.31	0.46	0.22	0.41	−0.18
	**Southern**	0.38	0.48	0.57	0.5	0.4	0.49	0.19	0.39	−0.19
	**Northern**	0.26	0.44	0.33	0.47	0.34	0.47	0.23	0.42	−0.03
**Shock 4**	**Central**	0.72	0.45	0.75	0.43	0.5	0.5	0.19	0.39	−0.53
	**Southern**	0.79	0.41	0.74	0.44	0.45	0.5	0.16	0.37	−0.63
	**Northern**	0.52	0.5	0.78	0.42	0.44	0.5	0.27	0.45	−0.25
**Shock 5**	**Central**	0.1	0.29	0.09	0.29	0.05	0.23	0.07	0.26	−0.03
	**Southern**	0.09	0.28	0.1	0.29	0.07	0.25	0.06	0.24	−0.03
	**Northern**	0.08	0.27	0.07	0.26	0.06	0.23	0.06	0.24	−0.02
**Shock 6**	**Central**	0.14	0.35	0.16	0.37	0.16	0.37	0.21	0.41	0.07
	**Southern**	0.13	0.34	0.15	0.36	0.16	0.36	0.18	0.38	0.05
	**Northern**	0.09	0.28	0.09	0.29	0.07	0.26	0.06	0.23	−0.03
**Shock 7**	**Central**	0.02	0.13	0.74	0.44	0.48	0.5	0.32	0.47	0.3
	**Southern**	0.02	0.14	0.64	0.48	0.6	0.49	0.28	0.45	0.26
	**Northern**	0.01	0.09	0.63	0.48	0.65	0.48	0.34	0.47	0.33

Notes:

Shock 1 : Death or serious illness of an adult member or someone who provides support for yourself or your family?

Shock 2: Poor crop yields, loss of crops due to disease or pests, or loss of livestock due to theft or disease, or loss of coupon?

Shock 3: Loss of source of income-such as loss of employment, business failure, someone who had been assisting the household stopped their support?

Shock 4: Price change in agriculture input or output?

Shock 5: Breakup of household, such as a divorce?

Shock 6: Damage to house due to fire, flood, or another unexpected event?

Shock 7: Any other shock (2008)? And Changes in crop yields (2010 and onward) Thus, we are not considering the last shock in our analysis in next section.

**Table 2:** Summary Statistics of proportion of boys and girls enrolled in school

										Diff of
		2008		2010		2017		2019		proportion
		Mean	S.D	Mean	S.D	Mean	S.D	Mean	S.D	2008–2019
**Prop. of boys enrolled (7–12)**	**Total**	0.76	0.19	0.77	0.19	0.89	0.18	0.83	0.20	0.07
	**Central**	0.76	0.21	0.74	0.22	0.91	0.21	0.82	0.26	0.06
	**Southern**	0.63	0.15	0.69	0.15	0.78	0.12	0.76	0.10	0.13
	**Northern**	0.83	0.14	0.84	0.14	0.93	0.15	0.89	0.13	0.06
**Prop. of boys enrolled (13–17)**	**Total**	0.68	0.25	0.72	0.22	0.75	0.28	0.73	0.26	0.05
	**Central**	0.66	0.29	0.72	0.26	0.71	0.32	0.69	0.29	0.03
	**Souther**	0.60	0.16	0.61	0.14	0.73	0.21	0.67	0.14	0.07
	**Northern**	0.74	0.22	0.77	0.18	0.83	0.24	0.81	0.23	0.07
**Prop. of girls enrolled (7–12)**	**Total**	0.74	0.20	0.74	0.21	0.86	0.24	0.82	0.21	0.08
	**Central**	0.71	0.23	0.71	0.25	0.86	0.26	0.81	0.24	0.10
	**Southern**	0.61	0.15	0.64	0.20	0.79	0.24	0.81	0.14	0.20
	**Northern**	0.85	0.13	0.82	0.13	0.88	0.21	0.87	0.19	0.02
**Prop. of girls enrolled (13–17)**	**Total**	0.70	0.24	0.69	0.26	0.70	0.31	0.68	0.25	−0.02
	**Central**	0.70	0.28	0.67	0.31	0.66	0.36	0.64	0.28	−0.06
	**Southern**	0.54	0.19	0.65	0.16	0.63	0.18	0.63	0.18	0.09
	**Northern**	0.76	0.17	0.74	0.22	0.80	0.26	0.77	0.21	0.01

**Table 3:** Fixed effect estimates (Dep Var: Proportion of boys enrolled at village level in the age group (7–12))

	(1)	(2)	(3)	(4)	(5)	(6)	(7)
**shock01**	−.115*						−.06
	(.063)						(.07)
**shock02**		.029					.114*
		(.06)					(.066)
**shock03**			−.053				.039
			(.062)				(.074)
**shock04**				−119***			−134***
				(.036)			(.041)
**shock05**					−.203		−.155
					(.146)		(.148)
**shock06**						.149	.122
						(.109)	(.109)
**_cons**	.665***	.609***	.653***	.703***	.646***	.609***	.624***
	(.095)	(.104)	(.097)	(.095)	(.094)	(.095)	(.105)
**sigma_u**	0.130	0.129	0.129	0.128	0.126	0.129	0.131
**sigma_e**	0.186	0.187	0.187	0.184	0.187	0.187	0.184
**Rho**	0.326	0.323	0.321	0.325	0.313	0.324	0.337
**Observations**	445	445	445	445	445	445	445

Standard errors are in parentheses

*** *p*<01

** *p*<.05

* *p*<.1

**Table 4:** Fixed effect estimates (Dep Var: Proportion of boys enrolled at village level in the age group (13–17))

	(1)	(2)	(3)	(4)	(5)	(6)	(7)
**shock01**	−.129*						−.119
	(.074)						(.081)
**shock02**		.106					18**
		(.07)					(.079)
**shock03**			−.026				−.004
			(.074)				(.087)
**shock04**				−.048			−.061
				(.044)			(.05)
**shock05**					−.14		−.096
					(.171)		(.177)
**shock06**						.253**	.249**
						(.123)	(.124)
**_cons**	.685***	.567***	.658***	.676***	.657***	.61***	.557***
	(12)	(.129)	(.122)	(.121)	(.119)	(.119)	(.131)
**sigma_u**	0.164	0.163	0.165	0.164	0.164	0.171	0.168
**sigma_e**	0.235	0.235	0.236	0.235	0.236	0.234	0.233
**Rho**	0.329	0.325	0.328	0.326	0.325	0.346	0.342
**Observations**	464	464	464	464	464	464	464

Standard errors are in parentheses

*** *p*<.01

** *p*<.05

* *p*<.1

**Table 5:** Fixed effect estimates (Dep Var: Proportion of girls enrolled at village level in the age group (7–12))

	(1)	(2)	(3)	(4)	(5)	(6)	(7)
**shock01**	−.03						.082
	(.07)						(.075)
**shock02**		.049					.189***
		(.064)					(.069)
**shock03**			−.199***				−.163**
			(.067)				(.078)
**shock04**				−.149***			−159***
				(.039)			(.044)
**shock05**					−.216		−.092
					(.156)		(.156)
**shock06**						.226**	.2*
						(.115)	(.113)
**_cons**	.359***	.313***	.432***	.441 ***	.367***	.318***	.326***
	(.108)	(.116)	(.108)	(.106)	(.106)	(.106)	(.115)
**sigma_u**	0.145	0.148	0.143	0.145	0.147	0.150	0.155
**sigma_e**	0.211	0.211	0.208	0.206	0.210	0.210	0.203
**rho**	0.322	0.329	0.322	0.330	0.327	0.337	0.368
**Observations**	455	455	455	455	455	455	455

Standard errors are in parentheses

*** *p*<.01

** *p*<.05

* *p*<.1

**Table 6:** Fixed effect estimates (Dep Var: Proportion of girls enrolled at village level in the age group (13–17))

	(1)	(2)	(3)	(4)	(5)	(6)	(7)
**shock01**	.065						.037
	(.08)						(.087)
**shock02**		.093					.051
		(076)					(.086)
**shock03**			.086				.059
			(.081)				(.097)
**shock04**				.054			.031
				(.048)			(.055)
**shock05**					−.195		−.264
					(.197)		(.203)
**shock06**						.185	.206*
						(.122)	(.122)
**_cons**	.402***	.351**	.386***	.388***	.436***	.395***	.319**
	(.129)	(.139)	(.131)	(.13)	(.128)	(.128)	(.142)
**sigma_u**	0.167	0.167	0.166	0.166	0.166	0.172	0.175
**sigma_e**	0.254	0.254	0.254	0.254	0.254	0.253	0.254
**rho**	0.301	0.303	0.300	0.301	0.299	0.316	0.322
**Observations**	462	462	462	462	462	462	462

Standard errors are in parentheses

*** *p*<.01

** *p*<.05

* *p*<.1

**Table 7:** Summarized table of effects of shocks on different cohorts

	Prop. of boys enrolled (7–12)	Prop. of boys enrolled (13–17)	Prop. of girls enrolled (7–12)	Prop. of girls enrolled (13–17)
Shock 1	−ve	−ve	−	−	
Shock 2	−	−	−	−	
Shock 3	−	−	−ve	−	
Shock 4	−ve	−	−ve	−	
Shock 5	−	−	−	−	
Shock 6	−	+ve	+ve	−	

## Footnotes

[1] National Statistics Office of Malawi

[2] The first wave of the MLSFH was funded by the Rockefeller Foundation. Subsequent funding has been provided by the NICHD (grants R01 HD053781, R01 HD/MH041713, R01 HD37276, R01 HD044228, R21 HD050652, R03 HD058976, R21 HD071471), and has been supported by pilot grants from the Population Studies Center (PSC), Population Aging Research Center (PARC), the Boettner Center for Pensions and Retirement Security, the Institute on Aging, and the Center for AIDS Research (CFAR), all at the University of Pennsylvania and supported among other sources by NIH grants NICHD R24 HD044964, NIA P30 AG12836, NIAID AI 045008. The project also received funding from the University of Pennsylvania Research Foundation.

[3] See for instance, Becker and Tomes (1986), Beegle et al, (2006a), and Fitzsimons and Mesnard (2008)
